# Supplementary material for: Genetic associations with educational fields
Source: Nat Genet. 2025 Nov 4;57(12):2997–3006. doi: 10.1038/s41588-025-02391-z (PMC12695639; doi:10.1038/s41588-025-02391-z)
Supplement: Supplementary file 1 — Supplementary Figs. 1–32 and Notes. [file 41588_2025_2391_MOESM1_ESM.pdf]

nature genetics

Supplementary information https://doi.org/10.1038/s41588-025-02391-z

Genetic associations with educational fields

In the format provided by the authors and unedited

SUPPLEMENTARY INFORMATION FOR CHEESMAN ET AL. GENETIC ASSOCIATIONS WITH EDUCATIONAL FIELDS.

Supplementary Figures 1-20: Manhattan and QQ plots from educational field GWA meta-analyses for fields with genome-wide significant findings 2

Supplementary Figure 21 and accompanying note: Causal relationships between educational fields and educational attainment 12

Supplementary Figures 22 and 23: Spread of educational attainment per field in MoBa 14

Supplementary Figure 24: Gene-environment correlation processes involved in genetic associations with educational field choices 15

Supplementary Figure 25: Parallel analysis 16

Supplementary Figure 26: The genetic structure of educational fields without controlling for educational attainment 17

Supplementary Figures 27-28: Manhattan plots for PCs 1 and 2, respectively 18

Supplementary Figure 29: Fields of study by sex for genotyped individuals in MoBa 19

Supplementary Figure 30: PCA of genetic correlations among the 7 most sex-balanced educational fields 20

Supplementary Figures 31-32: PCA of genetic correlations among the 10 educational fields in MoBa, split by sex 21

Supplementary Note: Frequently Asked Questions (FAQ) for "Genetic associations with educational fields" 23

Supplementary Note: Ethical Approvals 30

Supplementary Figures 1-20: Manhattan and QQ plots from educational field GWA meta-analyses for fields with genome-wide significant findings

Education (no EA adjustment); sum of effective sample sizes = 102,970:

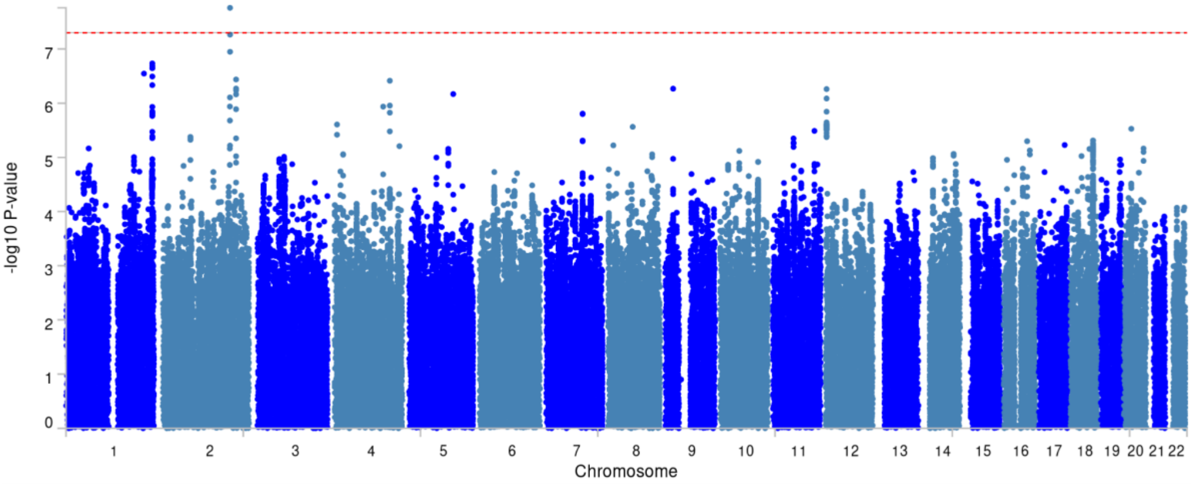

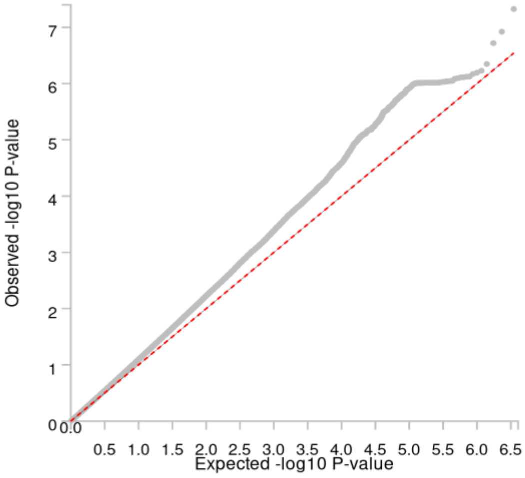

Arts and humanities (no EA adjustment); sum of effective sample sizes = 97262:

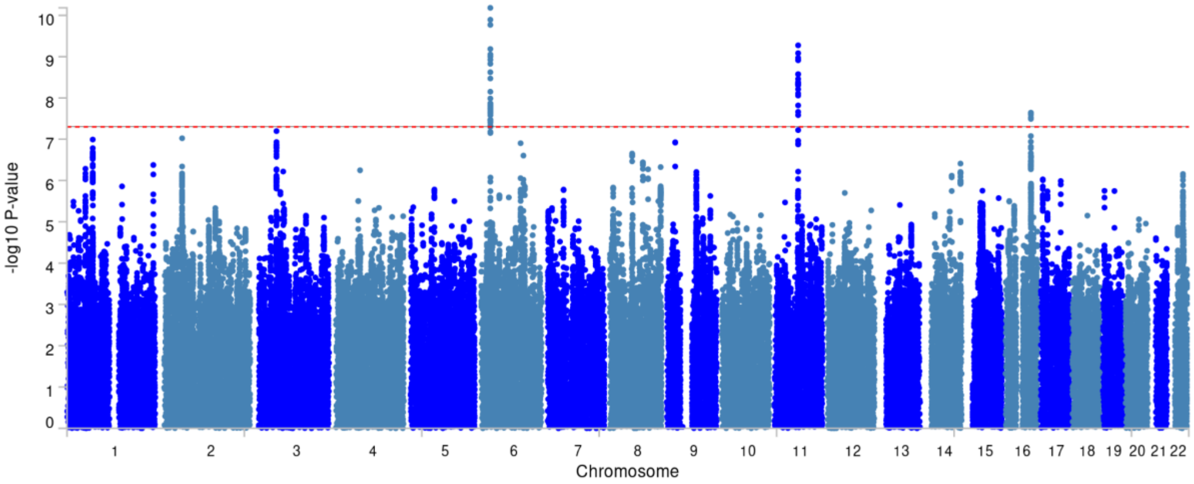

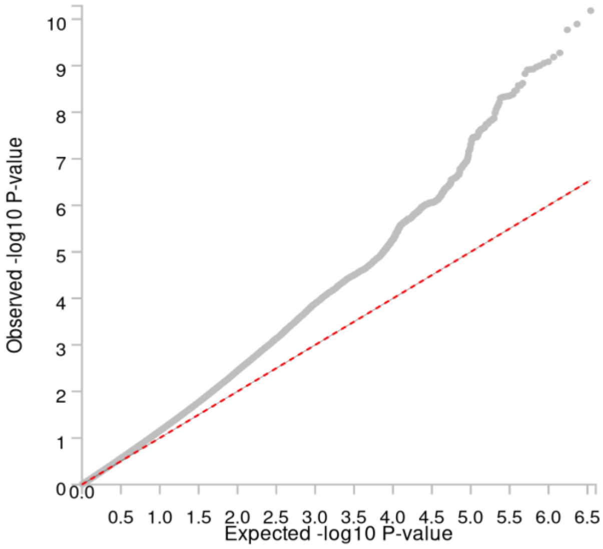

Social sciences and journalism (no EA adjustment); sum of effective sample sizes = 69123:

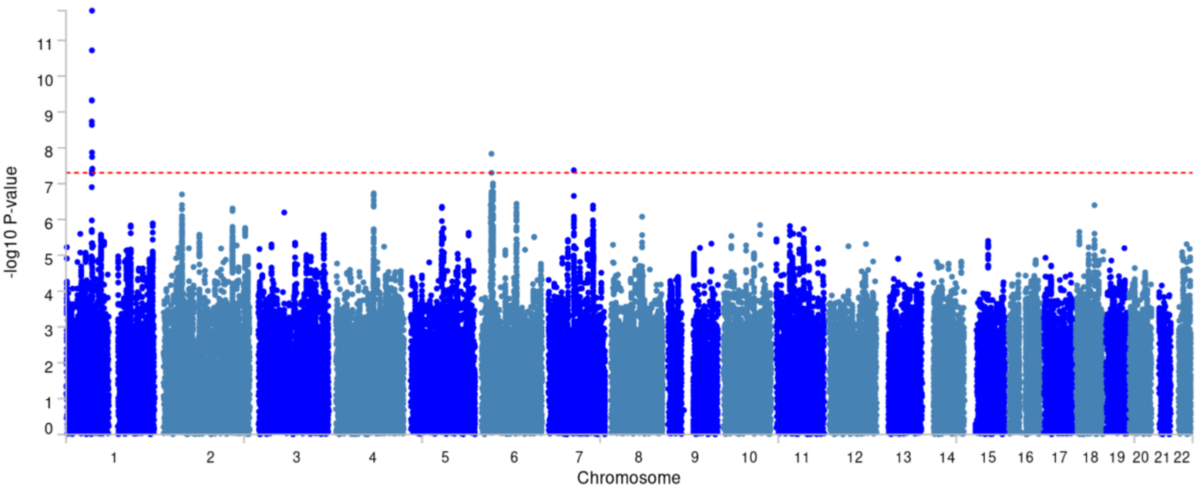

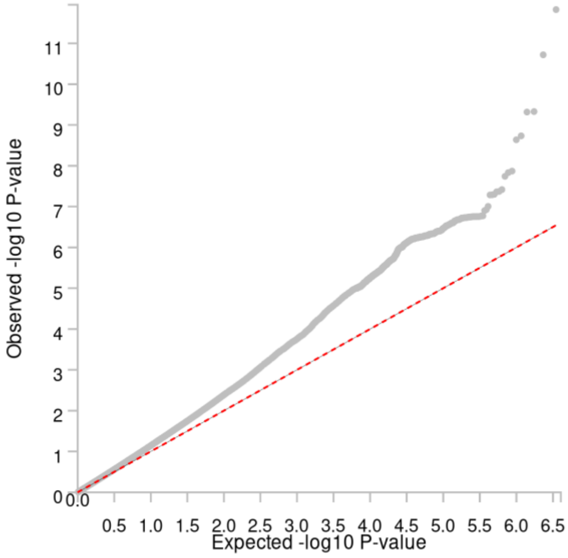

Business administration law (no EA adjustment); sum of effective sample sizes = 261,182:

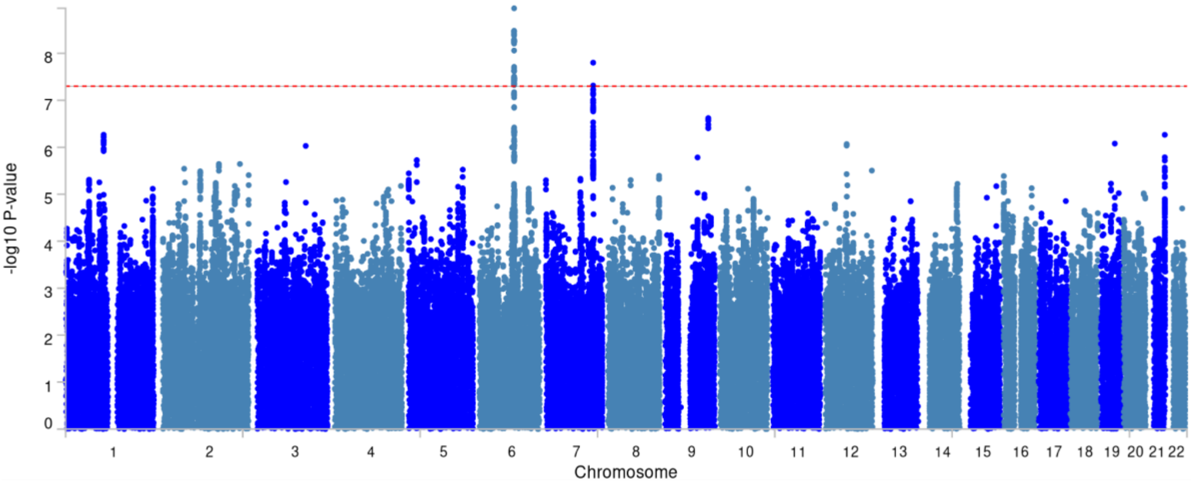

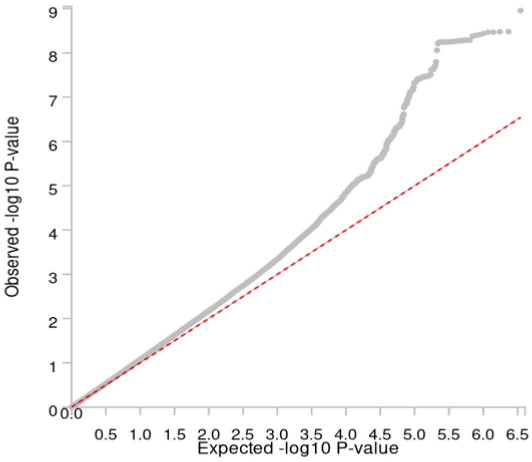

Natural sciences mathematics (no EA adjustment); sum of effective sample sizes = 40,072:

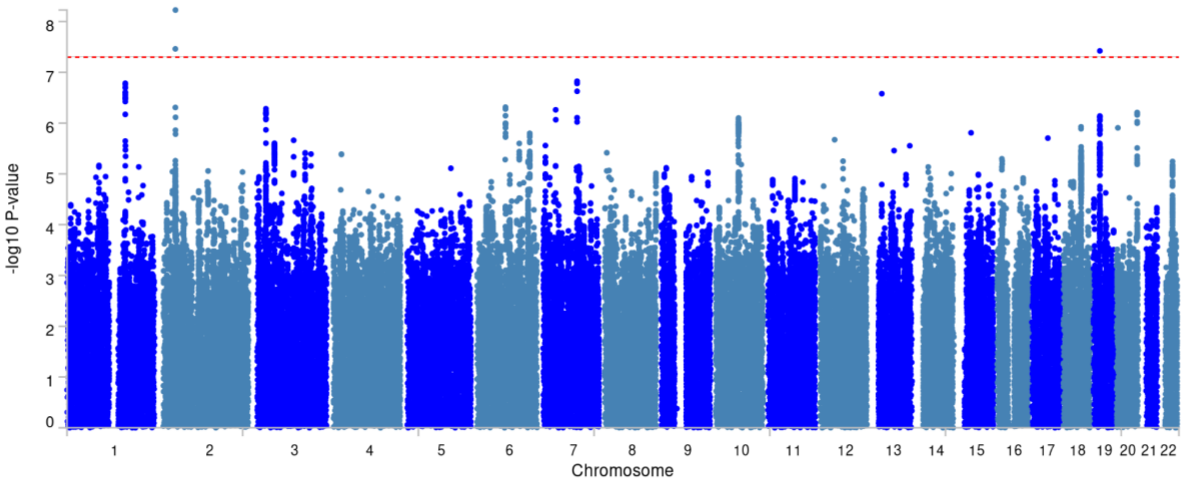

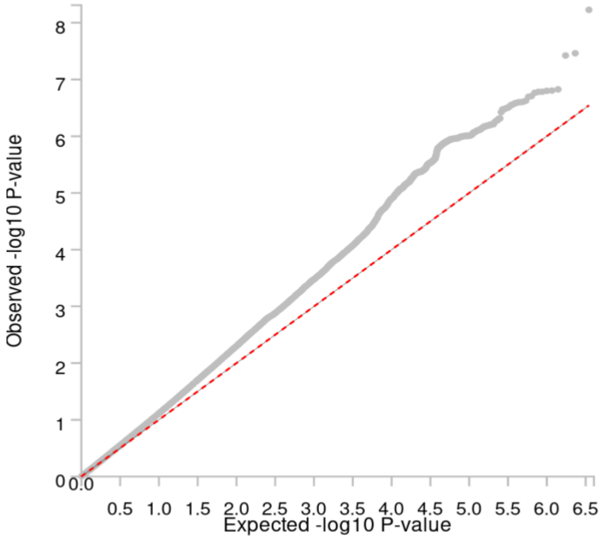

Natural sciences mathematics (**with** EA adjustment through GWAS by subtraction):

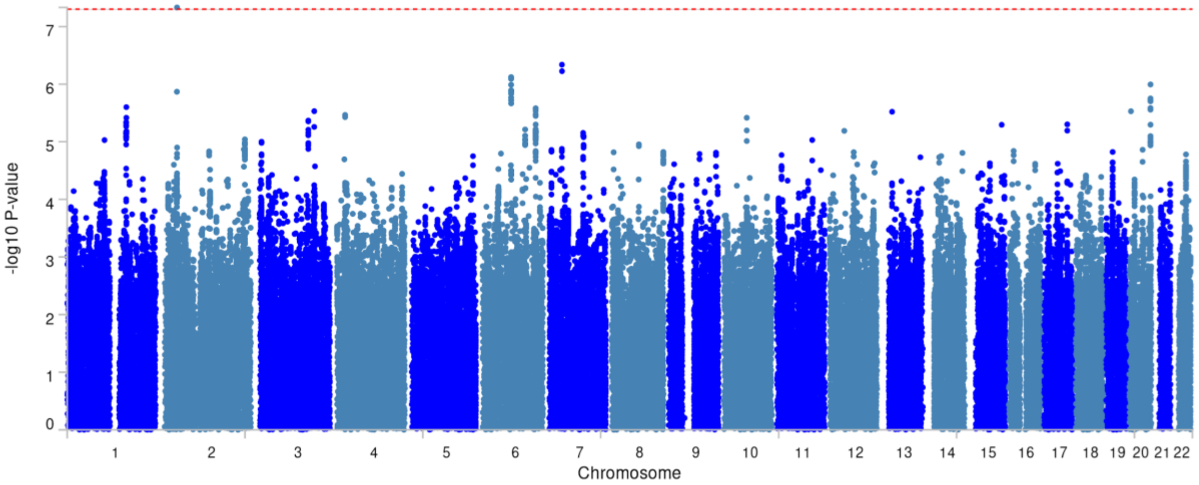

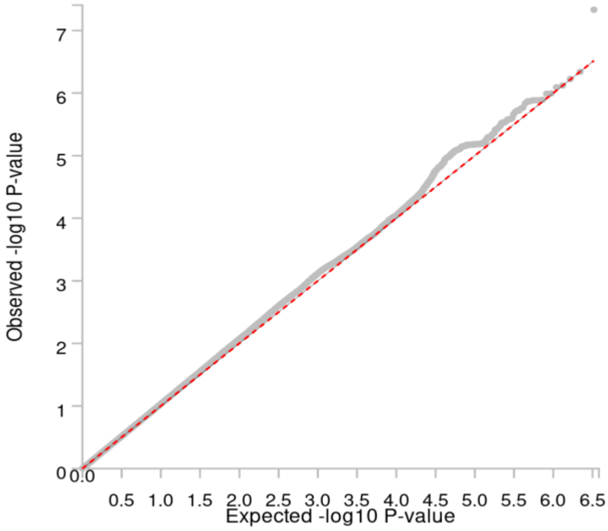

Engineering manufacturing construction (no EA adjustment); sum of effective sample sizes = 317,209:

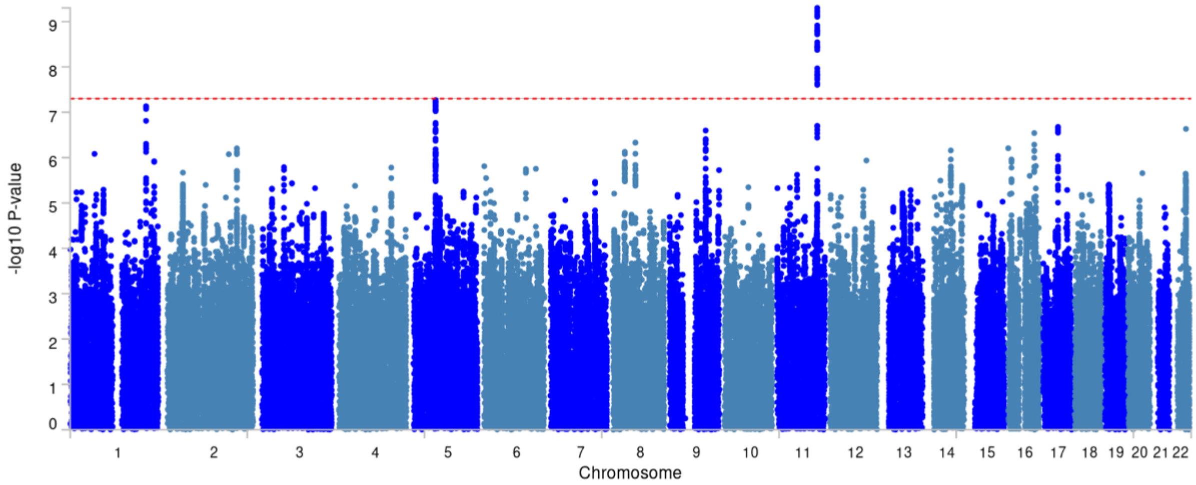

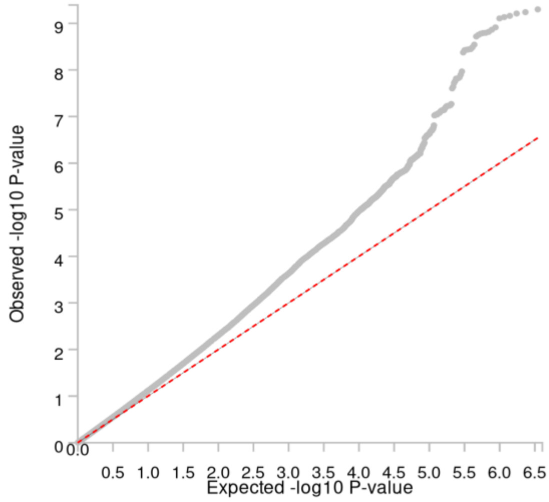

Engineering manufacturing construction (**with** EA adjustment through GWAS by subtraction):

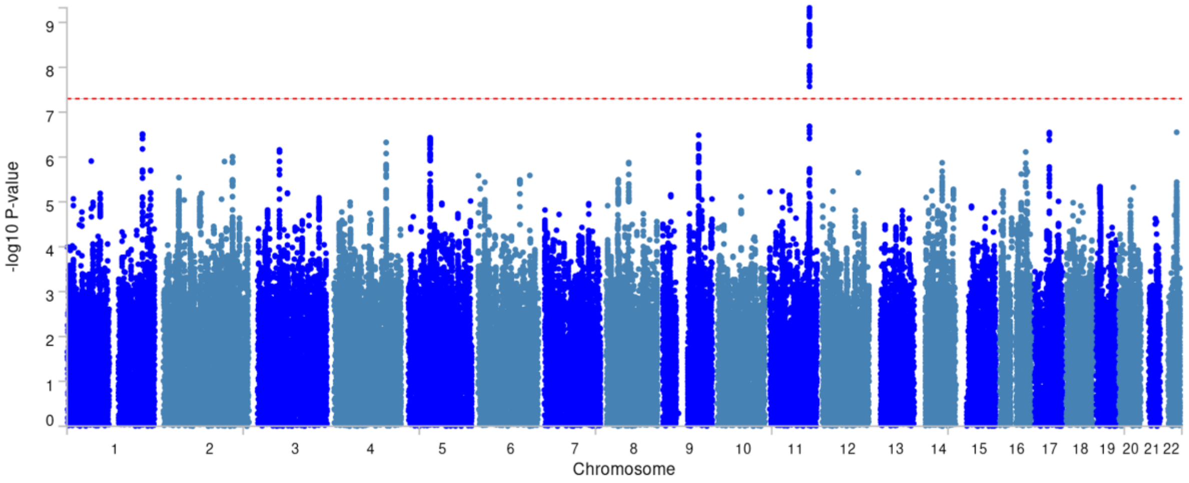

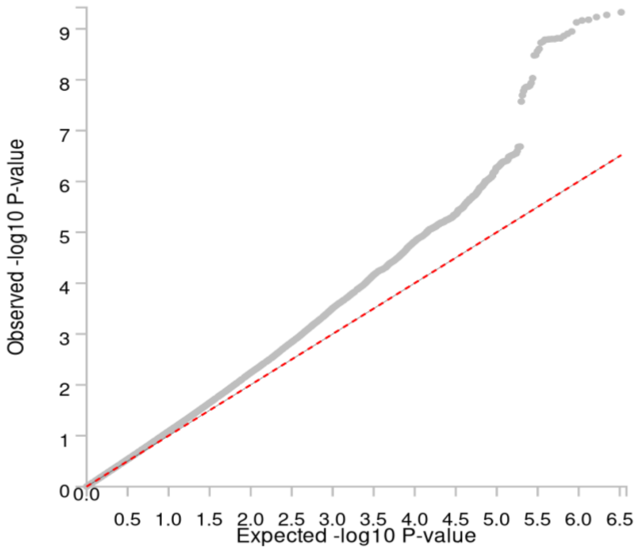

Health and welfare (no EA adjustment); sum of effective sample sizes = 292,929:

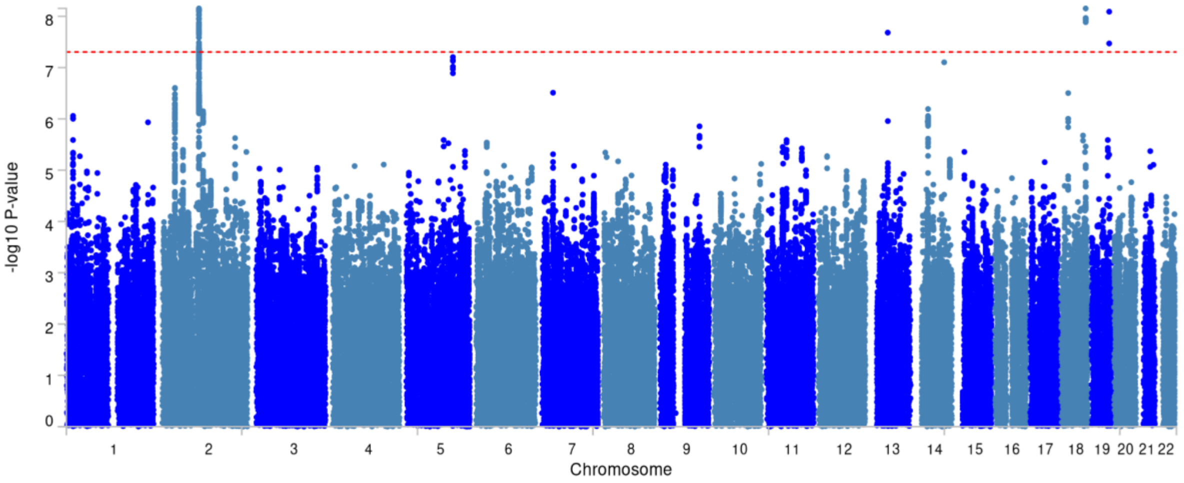

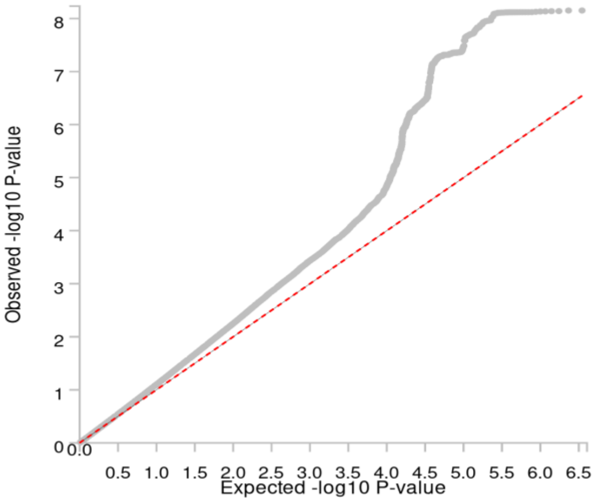

Health and welfare (**with** EA adjustment through GWAS by subtraction):

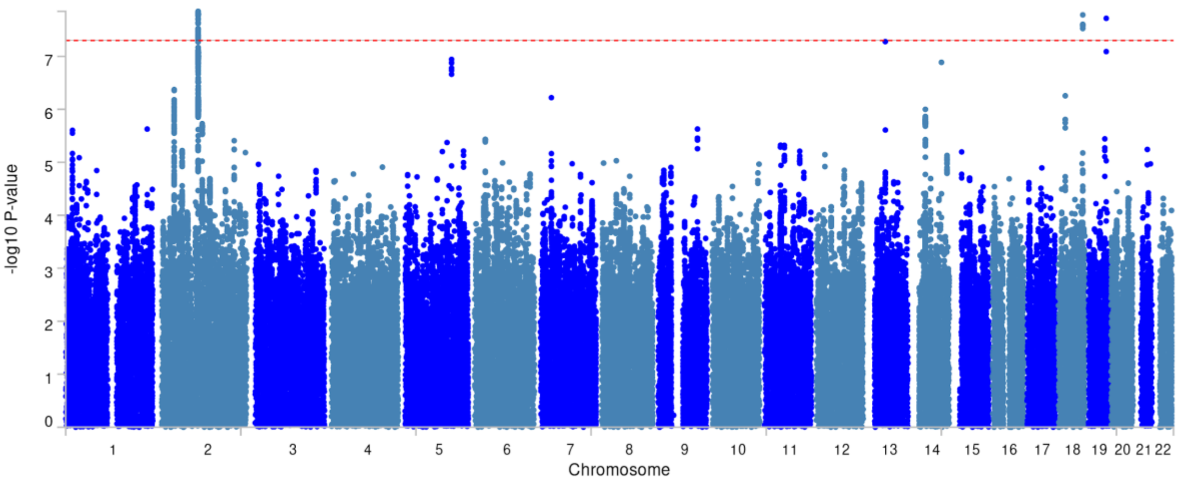

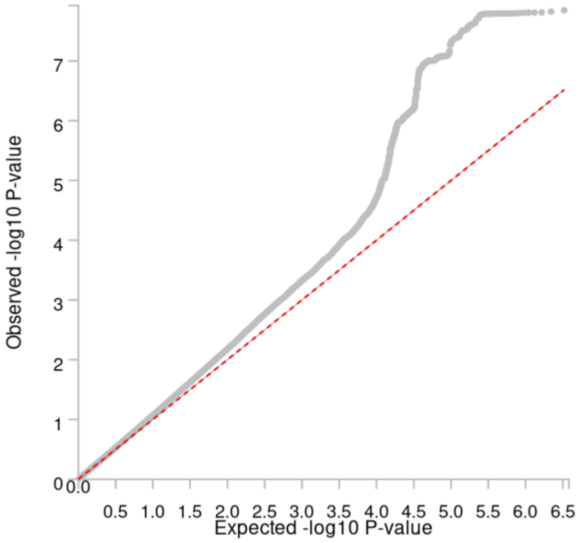

Supplementary Figure 21 and accompanying note: Causal relationships between educational fields and educational attainment

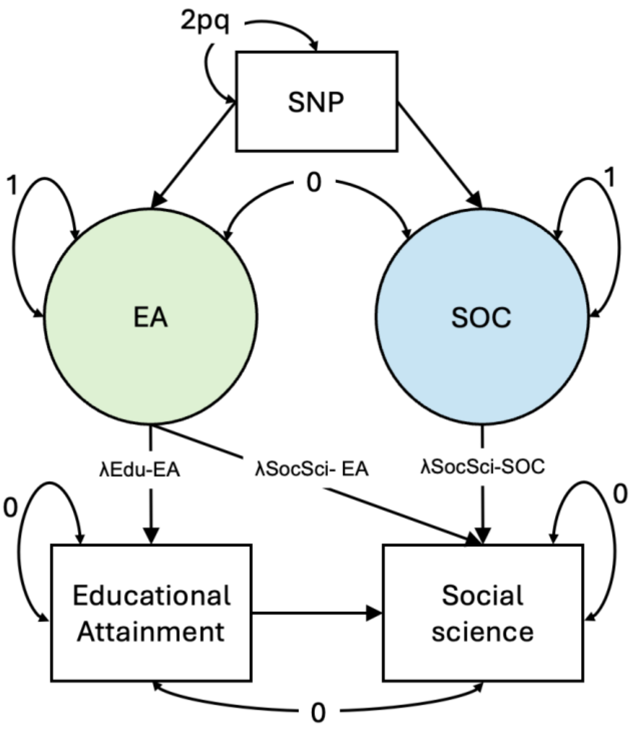

There are multiple possible causal relationships between educational field and educational level, and these might differ between fields.

Educational attainment is likely to be a consequence of field choice, especially since individuals often choose their field before finishing their education. For example, a person with a strong interest in languages might seek out a higher-level qualification to strengthen their skills.

However, the reverse is also possible. Individuals might choose a field based on the expected duration of their education and their preference for this duration. This could be based on a general tendency to seek out academic challenges and/or labour market returns. For example, a person who performs well at school might choose a programme like social sciences that is more likely to lead to a longer period of studying and learning.

Controlling for EA: strategy and assumptions of the current study
Our study emphasises the GWAS results where educational attainment was not controlled for. This enables us to avoid making assumptions about causal direction of effects between educational attainment and educational fields, and because we avoid the potential problem that educational attainment acts as a collider.
Nonetheless, it was necessary to control for educational attainment a) to explore horizontal stratification, which, by definition, refers to distinctions *within* any given educational level, and b) to explore vocational interests, which are better captured when holding educational level constant.

It is artificial to separate preference for a field and preference for a duration as the education system combines these two. When a person chooses a study programme, they are choosing a field *and* level. Medical doctors usually have long education and so attainment is an intrinsic part of the choice. Selection into different combinations of levels and fields – such as nursing being a Tertiary-level degree within the health field – will vary across educational programs available in the system.

They will also vary across countries and birth cohorts as educational systems and opportunities change over time. It may therefore be unrealistic that any given statistical procedure will remove all variance that is due to the level of qualification people have. Our separation of these is an abstraction that we impose on the "choice architecture" embedded in the educational system. We implement the following methods to control for educational attainment at the SNP level:

i) GWAS-by-subtraction

The GWAS-by-subtraction model for the example of social science, with path estimates for a single SNP, is shown in the Supplementary Figure above. SNP, and educational attainment and *Social science* are observed variables based on GWAS summary statistics. The genetic covariance between these is estimated based on their GWAS summary statistics. The model is fitted to a 3 × 3 observed variance–covariance matrix (that is, SNP, educational attainment, social science). EA and SOC are latent (unobserved) variables. The covariances between the educational attainment and social science variables are fixed to 0. The variance of the SNP is fixed to the value of 2pq (p = reference allele frequency, q = alternative allele frequency, based on 1000 Genomes Project phase 3). The residual variances of EA and SOC are fixed to 0, so that all variance is explained by the latent factors. The variances of the latent factors are fixed to 1. The observed variables were regressed on the latent variables, resulting in the estimates for the path loadings: λEdu-EA=0.37; λSocSci- EA= 0.28; λSocSci-SOC=0.18. Though not fully realistic, we assume that EA causes field choice and that there is no path from the latent SOC factor to observed educational attainment. See Supplementary Table 7 for path loadings for the other fields. The latent variables were then regressed on each SNP that met quality control criteria.

Notably, we found that the GWAS-by-subtraction did not fully remove genetic overlap with educational attainment (EA) for all fields, as indicated by non-zero genetic correlations between several residual field factors and EA, as well as between PC2 and EA (Supplementary Table 8). The degree of genetic correlation with EA varies across fields (average 0.11, minimum = 0.0009 for Health, maximum= 0.3 for Social sciences). It may be problematic and rather circular to use EA summary statistics in the model, export 'subtracted' summary statistics and recalculate genetic correlations. Multiple issues with estimating SNP-level effects such as sample overlap and population stratification can compound and be reintroduced when re-estimating genetic correlations. This makes it difficult to interpret genetic correlations between EA and fields after GWAS-by-subtraction. Even if we do take the genetic correlations with EA to be accurate, they are considerably attenuated after GWAS-by-subtraction, and the genetic correlation with PC2 of 0.21 is not so large as to imply that PC2 simply captures EA.

ii) Phenotypic adjustment

We also compare the GWAS-by-subtraction approach to the approach of simply controlling for EA phenotypically. The advantage of the phenotypic approach is that we control for EA in the specific Norwegian and Finnish cohorts, whereas the EA-GWAS might not be fully overlapping in genetic influences. However, because educational attainment is influenced by non-genetic factors and because educational attainment is a coarse measure (it is discrete with just a few categories, whereas the genetic factor for educational attainment is essentially continuous), controlling for educational attainment using Genomic SEM and the >1m person EA-GWAS offers a more comprehensive control.

Supplementary Figures 22 and 23: Spread of educational attainment per field in MoBa

Histograms and boxplots below plot educational levels (Norwegian NUS categories). Converting to the ISCED system (US-based), the average number of years of education per field specialisation (in order) were: 15.4 Services, 16.1 Agriculture, 17.1 Business, administration and law, 17.7 Arts humanities and languages, 17.8 Health and welfare, 18.9 ICT, 19.1 Education, 19.4 Social sciences, journalism and information, and 19.7 Natural sciences mathematics and statistics. Variation in years of education ranged from 0.1 Education, 0.5 Social sciences, 0.8 Natural sciences, 2.2 ICT, 7.1 Health and welfare, 8.7 Services, 8.8 Engineering, 8.9 Arts and humanities, 9.2 Business, and 11.5 Agriculture.

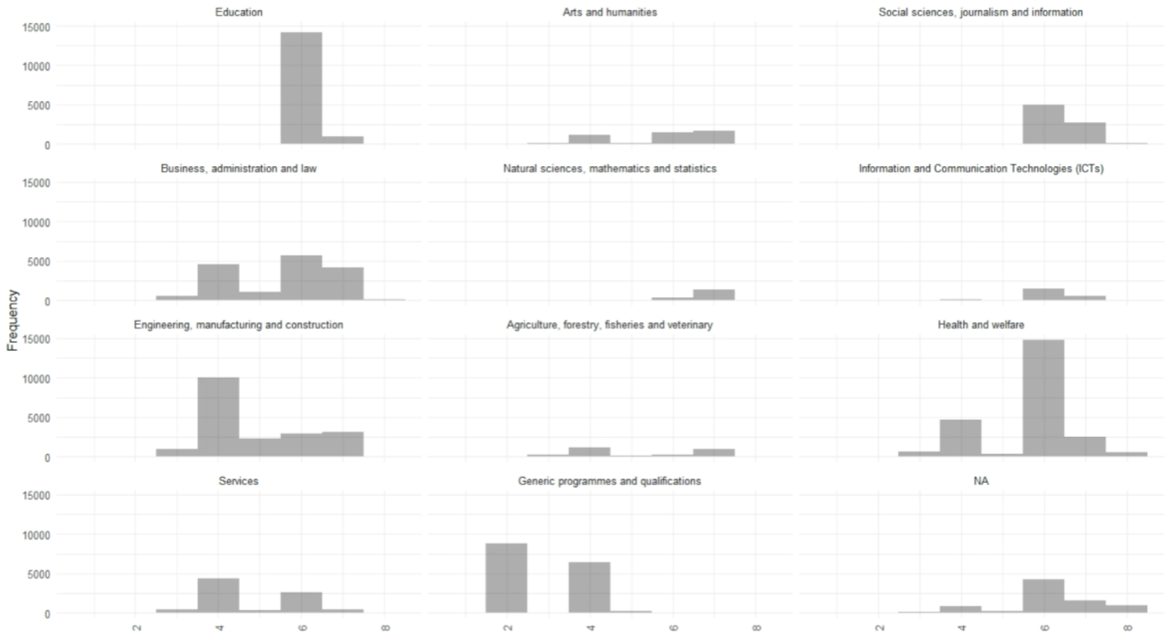

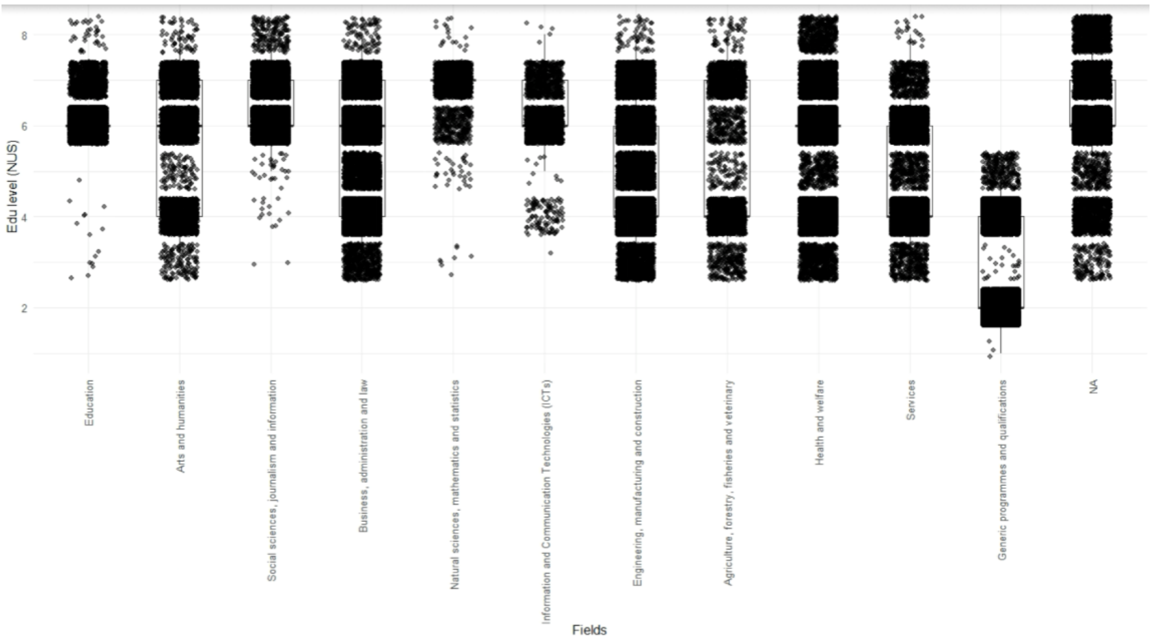

Supplementary Figure 24: Gene-environment correlation processes involved in genetic associations with educational field choices

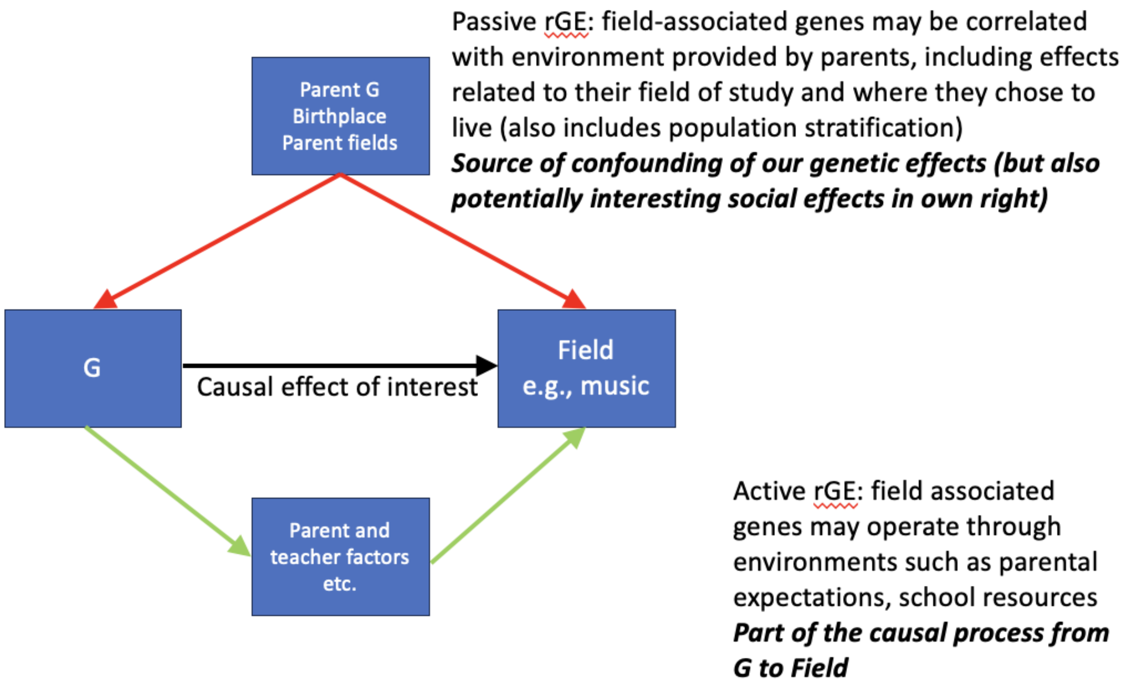

Note: rGE= gene-environment correlation.

Supplementary Figure 25: Parallel analysis

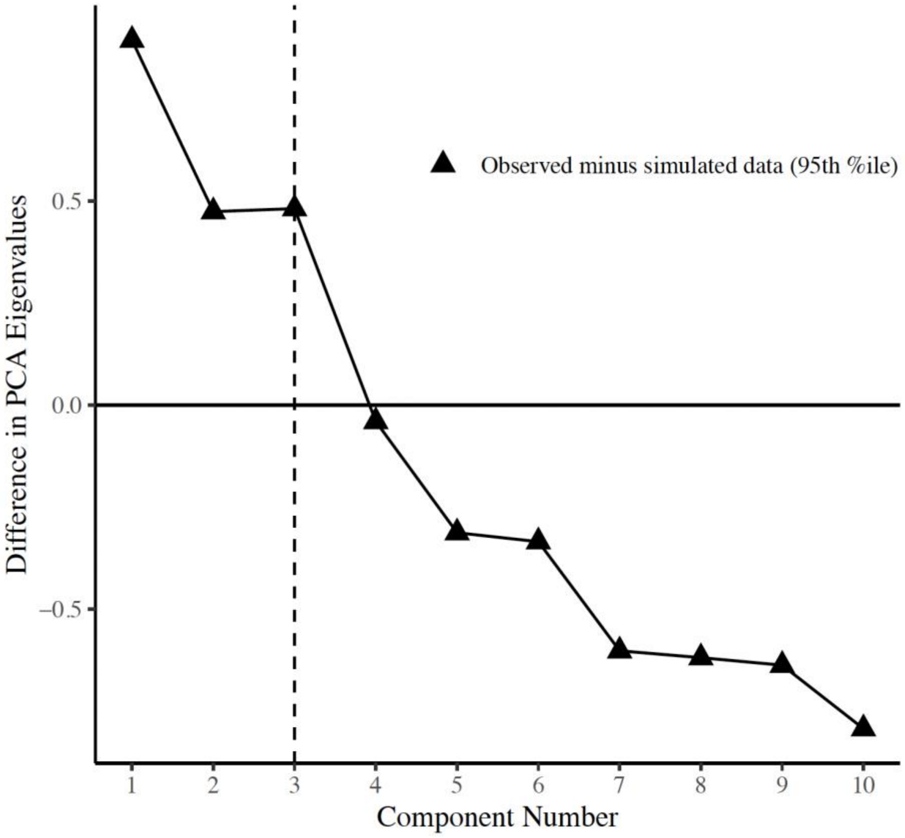

Supplementary Figure 26: The genetic structure of educational fields without controlling for educational attainment

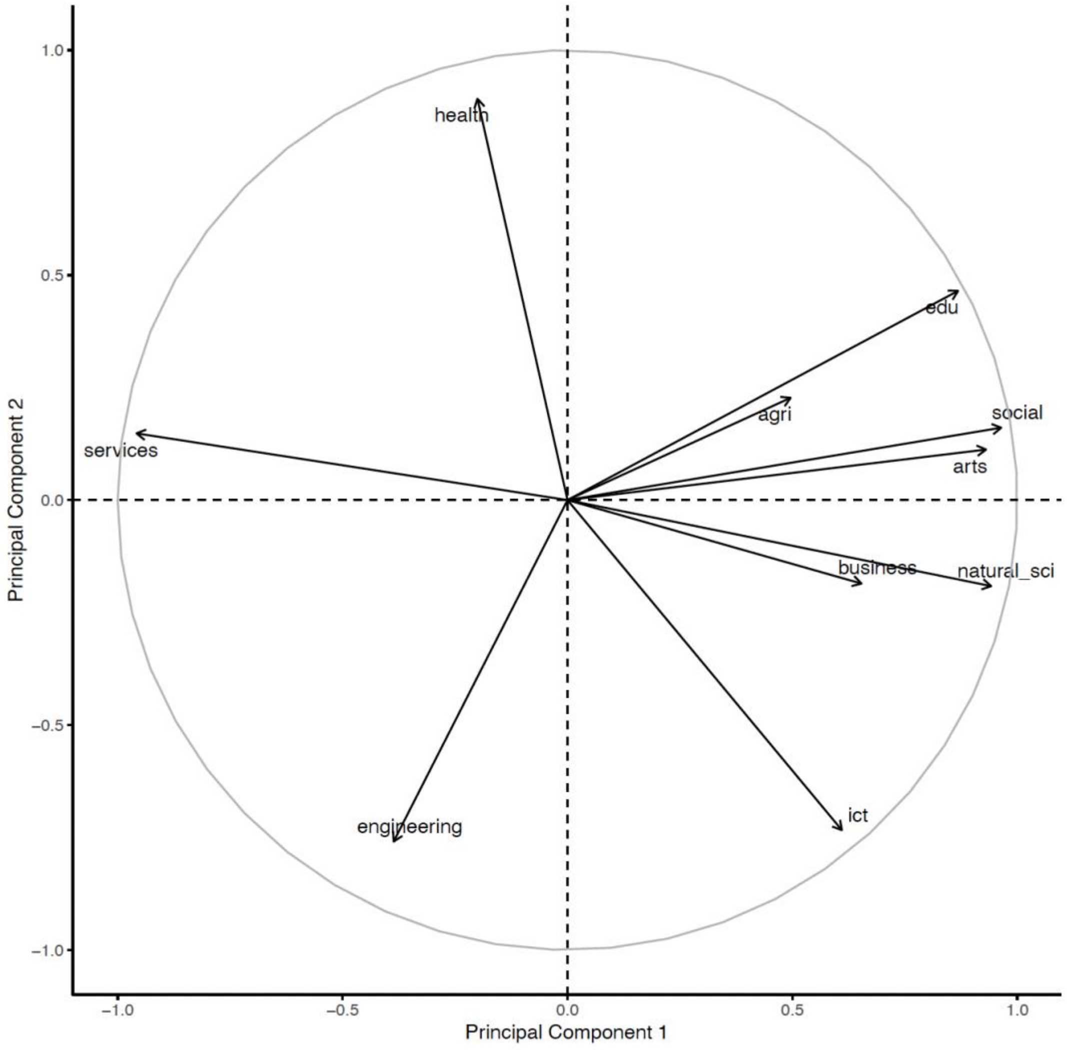

Supplementary Figures 27-28: Manhattan plots for PCs 1 and 2, respectively

PC1: Technical-Social *(effective N= 10413)*

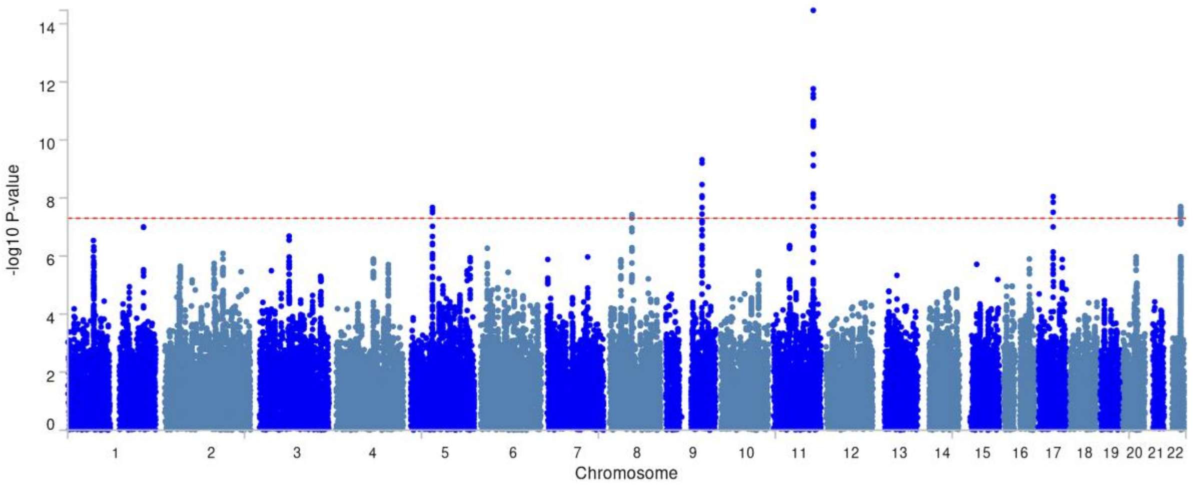

PC 2 Practical-Abstract (*effective N = 7353)*

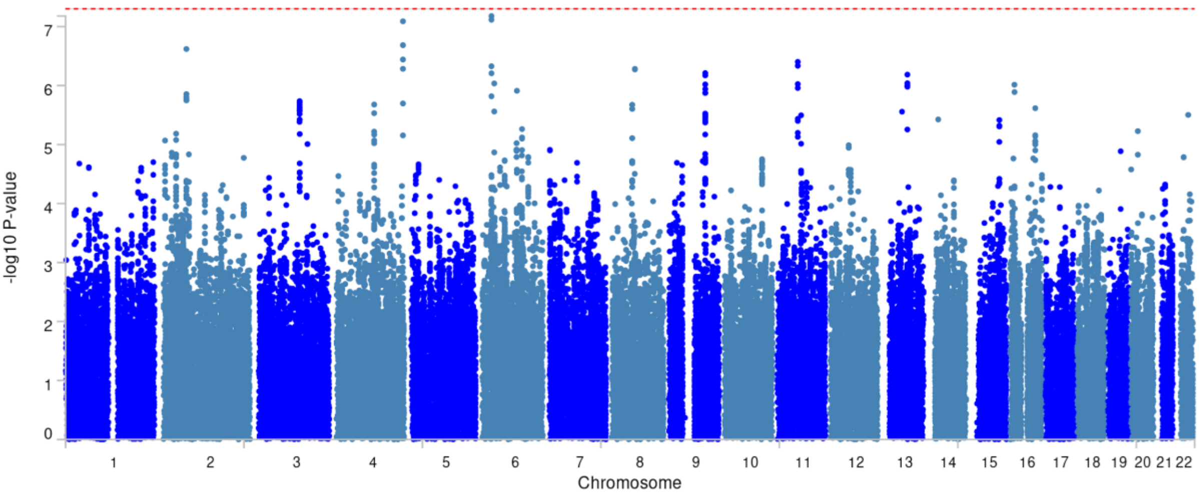

Supplementary Figure 29: Fields of study by sex for genotyped individuals in MoBa

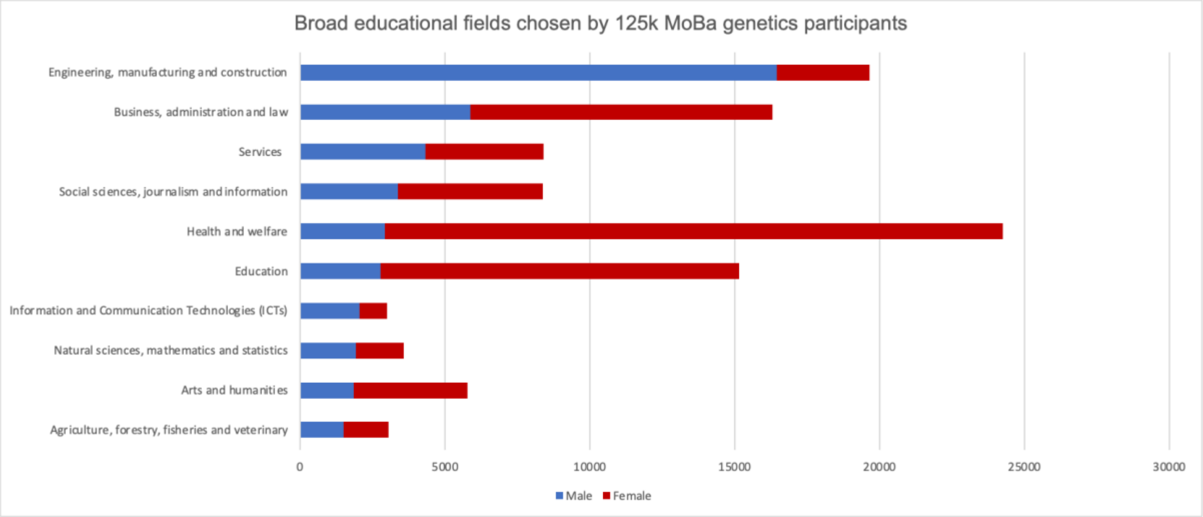

Supplementary Figure 30: PCA of genetic correlations among the 7 most sex-balanced educational fields

We performed PCA after removing fields that are most strongly stratified by sex. Like the main results including all 10 fields, we saw that 2 components explained most of the variance (69%). The first component again represents 'technical' subjects (ICT, natural science), whereas 'social' fields such as social sciences and services load negatively. The second component again represents 'abstract' fields in the Arts and humanities, with negative loadings for Services.

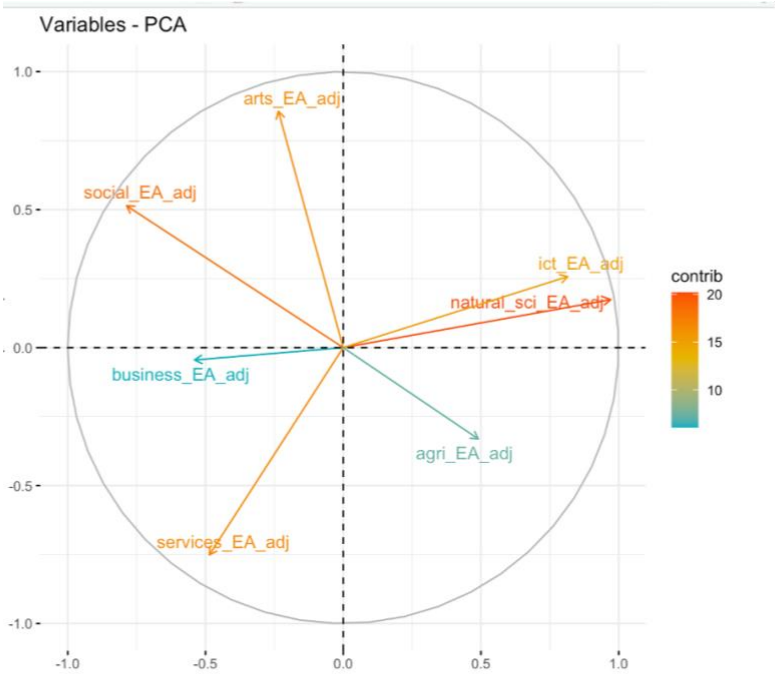

Supplementary Figures 31-32: PCA of genetic correlations among the 10 educational fields in MoBa, split by sex

As shown in the plot, sex-specific fields GWAS results contribute similarly to the genetic structure of the fields. Technical subjects like engineering contribute most to component 1, and abstract subjects like arts humanities and social sciences contribute most to component 2. Note that several sex-specific fields GWAS could not be included here due to inadequate power (education in men, agriculture in women, natural sciences in both). Results suggest that, even within sex, the technical versus social divide in genetic associations with field choices exists.

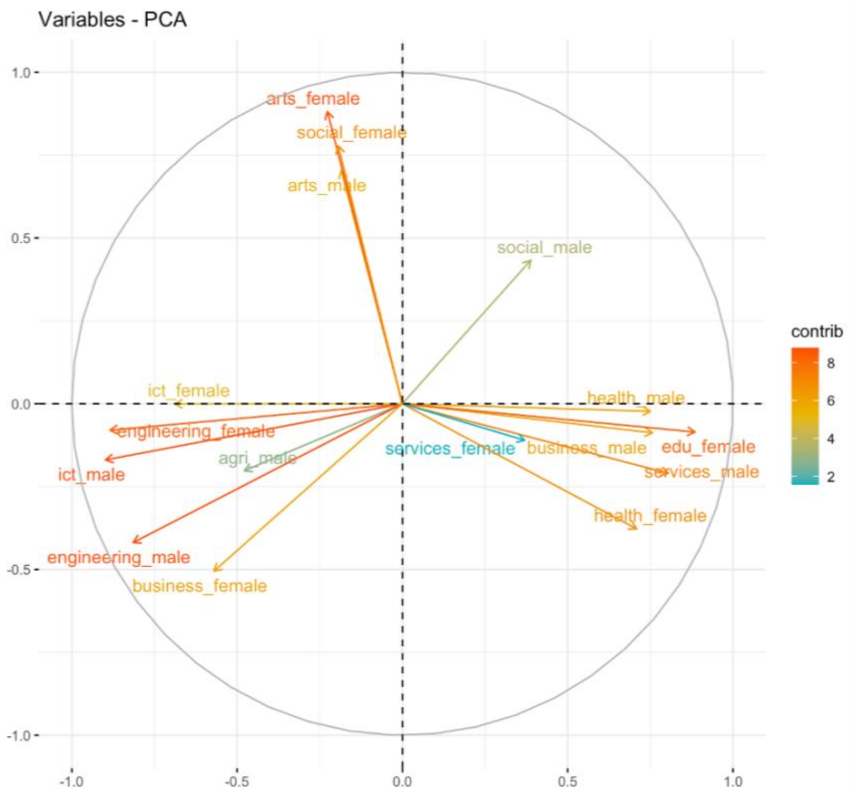

When we also repeat the analyses in males and females separately, we see the same pattern of technical subjects and arts subjects driving the first and second PC, respectively. Interestingly, the major axis of variation for men is ‘technical versus other choices’, and the major axis for women is ‘healthcare versus other choices’, which may reflect gender norms. However we are reluctant to over-interpret these results due to the small numbers of cases in most groups other than those with large differences in uptake by sex.

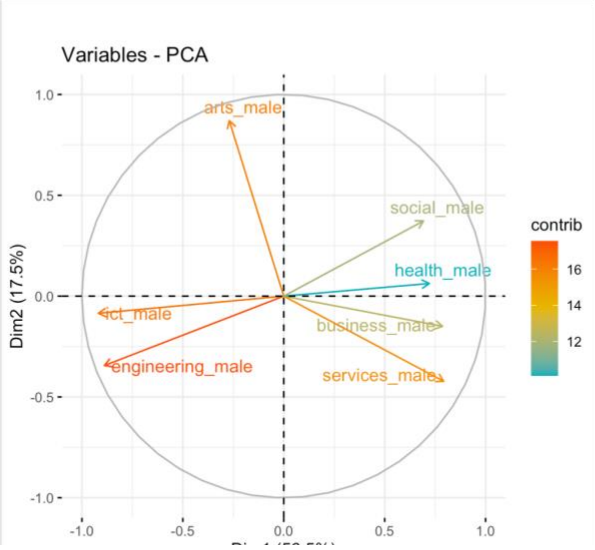

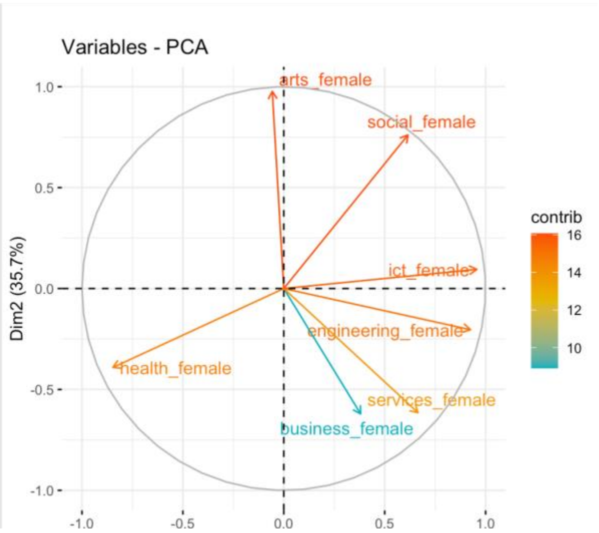

Supplementary Note: Frequently Asked Questions (FAQ) for "Genetic associations with educational fields"

By Rosa Cheesman. rosacg@uio.no

Box: Key terms

- **Educational fields** are domains of knowledge, skills, and competencies that are the subject matter of an education program, qualification, or degree. We study 10 broad fields of education defined by ISCED (International Standard Classification of Education). ISCED fields are standardized categories for classifying educational content at an international level, designed primarily for statistical reporting and comparison across different countries' education systems. We study educational field qualifications at all levels, not just school subjects or college majors. The focus is on educational qualifications not occupations or labour market sectors, which may align but are not identical to educational fields.
- A **genetic association** refers to a statistical relationship between a specific genetic variant (or multiple variants) and a particular trait or condition. When a genetic variant occurs more frequently in people with a certain characteristic than would be expected by chance, we say there is an association between that variant and the trait.
- The **GWAS (Genome-Wide Association Study)** is a research approach that examines millions of genetic variants across the human genome to identify which variants are statistically associated with a particular trait, disease, or condition. GWAS compares the genetic profiles of many individuals (in this study, 460,000) to detect variants that appear more frequently in people with the trait of interest compared to those without it.
- **Genetic variants** are differences in DNA sequences that occur among individuals. These are the natural variations in our genetic code that make each person genetically unique. We study the most common type of genetic variants known as Single Nucleotide Polymorphism (SNPs). Here, a single base pair (A, T, G, or C) in the DNA sequence differs from the usual base at that position. For example, a DNA sequence might read AAGGCT in most people, but AAGGTT in some individuals.
- **A polygenic index** (also called polygenic score or polygenic risk score) is a numerical value that summarizes the estimated effect of many genetic variants on an individual's phenotype (observable trait). It's calculated by summing the effects of multiple genetic variants, each weighted by the strength of its association with the trait. Polygenic indices are used to predict genetic predisposition to complex traits that are influenced by many genes, such as educational attainment, height, or disease risk.

What was the motivation for the study?

The choice of a field of study is one of the most profound decisions we can make. Field of education can significantly influence important outcomes like our income, and fertility, even when educational level is held constant. On a societal level, the systematic sorting of individuals into different fields shapes who acquires the skills specific employers want and thereby determines the distribution of rewards. Field-related social inequalities have been intensifying: as access to higher education expands, the specific area of study can increasingly influence life outcomes.

We know that social forces are important for field of study, and we also know that there is a genetic component to our behaviours and our educational trajectories. But what we don't know is **how genetic and social forces combine** to influence who is getting what qualifications. The aim of this project was to fill this gap. We realised that massive scale genetic data could be used to reveal novel and replicable information about the interests that drive us and the social inequalities that structure our lives.

Our goals were to:

- Explore whether genetic variants are associated with different *kinds* of qualifications, not just *levels* of qualifications.
- Identify clusters of fields that share a genetic basis. Individuals tend to only specialize in one field, making it difficult to understand similarities across fields. For example, we don't know whether there are any overlapping influences on choosing STEM versus Arts subjects. We use a new genetic method that can measure the similarity of two traits even if they are measured in two different groups.
- Describe the complexity of educational fields in terms of how they relate to myriad human traits, behaviours, diseases and positions in society. There has been some research on factors linked to educational fields (e.g., income, fertility), but we wanted to use genetic methods to expand the scope of the evidence and look at personality, mental and physical health, life satisfaction and more.

Who conducted this study?
The authors of the article are an interdisciplinary team of statistical geneticists, personality psychologists, economists and sociologists based in Norway, Finland and the Netherlands. Large scale collaboration is necessary for genome-wide association studies, which require large sample sizes to successfully identify genetic associations. The article bridges previously disconnected domains: the genetics of complex traits, on human interests and personality, and on educational sorting and inequality in society. This integration demanded diverse expertise from all coauthors, whose complementary specializations enabled us to develop more comprehensive theoretical frameworks and methodological approaches than would have been possible within a single discipline.

What did we do?
We investigated whether and how genetic factors are associated with what field people study, from fine art to finance.

We brought together a huge dataset of 460,000 genotyped people from across Finland and Norway and looked at the full range of qualifications they were recorded to have studied in the national educational registers. We followed international convention from the European Commission and looked at 10 broad field categories: *Education; Arts and humanities; Social sciences, journalism and information; Business, administration and law; Natural sciences, mathematics and statistics; Information and Communication Technologies; Engineering, manufacturing and construction; Agriculture, forestry, fisheries and veterinary; Health and welfare; Services*. The most common field codes were *Engineering, manufacturing and construction* and *Health and welfare*, whilst the least common were *Agriculture* and *Natural sciences, mathematics and statistics*.
The four main aspects of the study were:

1. To identify any links between genetic variants and educational fields, we used a well-established approach called a genome-wide association study (GWAS). GWAS allows us to scan across millions of genetic variants in the human genome to see if some occur statistically more often in people studying specific fields e.g., *Social sciences* than in people who did not specialise in that field. We did 10 genetic association studies (one for each field of education).
2. We then explored overlap in the genetic variants associated with *different* fields, e.g., to what extent do similar genetic variants play a role in specialising in *Natural sciences* and *Social sciences*?
3. To make the complex interrelationships between all the fields more interpretable, we applied another well-established method called principal component analysis to simplify our

genetic results. We identified a smaller number of key components to describe the main ways that people are sorted into groups of fields.
4. We explored in depth what the components of field qualifications mean by looking into how they correlate with ~100 other traits, behaviours, diseases and inequality indices analysed in external genetic studies.

We did lots of other analyses to validate and probe our findings, as detailed in the paper.

Who did we study and why does that matter?
Our study was based on cohorts from Finland and Norway. These countries have high-quality administrative records of their *whole populations'* educational pathways, enabling us to assemble a huge amount of data and harmonise it so that participants' age ranges and field categories match.

The Nordic context of our study is characterised by free education, universal stipends, and student loans with low interest rates to cover living expenses. This is an advantage when it comes to identifying genetic associations, because it increases the chances that we pick up on mechanisms involving genetically influenced interests and skills, rather than only non-genetic factors like tuition fees and family resources (although this signal is interesting too). Nonetheless, social inequalities still exist. Wage gaps between fields, and strong normative cultural beliefs about gender in Nordic countries also mean that our study does not purely capture individual interests.

The Nordic context does not necessarily generalise well. In countries with higher social inequality than in Finland and Norway (i.e., most countries!), where the socioeconomic consequences of some field choices are riskier, the heritability of field choices might be lower, and links with individual interests and preferences might be less prominent. Our results pertain to a specific cohort and socio-political context and would likely change along with changes to how people sort into fields. For example, the results might differ if people were encouraged to explore a wider range of subjects, if the skills involved in certain fields were different, or if the gender norms or economic returns to fields changed.

We also note the major limitation that only Norwegian and Finnish individuals with European-associated ancestries were included in the study. It remains unclear how much the results generalise to people of diverse ancestral backgrounds. Future work should include underrepresented groups and countries to increase generalisability and avoid reinforcing socioeconomic and health disparities.

With these caveats in mind, our study is a great starting point for understanding the role of genetics in educational field choices.

What did we find?

1. **Genetic differences between people are associated with differences in what field they study.**

Commonly occurring genetic variation in the population was found to be significantly associated for all the 10 fields. This is the first time genetic associations with fields have been shown. As with all complex human outcomes, each genetic variant has a miniscule effect on its own. To the extent that a given genetic variant influences educational field qualifications, it does so in combination with other variants and with environmental experiences. When we analyse all common genetic variants together, they capture between 3% (for Health and welfare) and 14% (for Natural sciences, mathematics and statistics) of the individual differences in educational field specialisations.

2. **There are underlying interrelationships between field specialisations.**

Even though each participant could only be observed in one category, we were able to demonstrate patterns of clustering between diverse educational fields using genetic data. For example, genetic differences linked to pursuing *Social sciences, journalism and information* are also strongly associated with studying *Arts and humanities* but not at all with studying *Natural sciences, mathematics and statistics.*

3. **Two key distinctions are important in how people sort into educational fields: Technical versus Social, and Practical versus Abstract.**

We extracted key components explaining the genetic clustering across fields. We label the first component 'Technical versus Social' because it indicates how much each field involves things (like bridges and numbers) versus people. *Engineering* is strongly Technical, whereas *Education* is on the Social end of the spectrum. We label the second component 'Practical versus Abstract' as it seems to reflect hands-on, pragmatic as opposed to theoretical and creative activities. More Practical fields include *Services* and *Health and welfare*, whereas more Abstract fields include *Social sciences, journalism and information, Natural sciences, mathematics and statistics,* and *Arts, humanities and languages.*

Interestingly, the structure behind different educational pathways that we elucidated chimes with social science theories. This includes not only the well-known RIASEC vocational interests model used by careers advisers to help people choose their life path, but also sociological theory on the key resources that specialised educational programmes provide. This striking alignment between social science theory and number crunching of massive genetic data shows how genetic differences reflect the social structure, and how genetic research complements social science enquiry.

4. **Genes linked to Technical-Social and Practical-Abstract field components are also associated with many sorts of domains including personality, mental health, substance use, health, fertility, and socioeconomic status.**

Genes don't operate in a vacuum but are expressed via interplay with our environments throughout our lives. As a result, genetic associations are a microcosm of all the psychological, social, geographical and cultural factors linked to field specialisations. To bring out these patterns, we examined how Technical-Social and Practical-Abstract qualifications link to ~100 traits.

Some of the results reflect plausible psychological mechanisms. The Technical-Social component is genetically correlated with traits that arise early in life and relate to being interested in people, such as extraversion, agreeableness (higher on the Social end). Similarly, this component overlaps genetically with non-cognitive skills (also known as 'socioemotional' skills), frequency of family/friend visits, and number of sexual partners. The Abstract-Practical component also relates to individual tendencies towards open personality and creativity. The tendency to study Abstract rather than Practical educational fields is genetically correlated with higher predisposition to schizophrenia and bipolar disorder, consistent with studies showing that relatives of people with these mental health conditions are more likely to have creative professions.

Our results also appear to capture wider patterns of social stratification. The Abstract-Practical component is clearly related to traditional socioeconomic indicators such as occupational status. Unlike Practical fields like education and healthcare, which are oriented towards welfare state jobs, Abstract qualifications often lead to elite professions in media, politics, research, law, and the arts,

which are typically more accessible to individuals from advantaged families. Therefore, our genetic findings might be capturing social and economic resources.

Interestingly, our genetic results paint a more nuanced picture of social and economic variation in the population than is possible when only looking at conventional status markers. It appears we may also be identifying some disadvantages of elite educational paths. For example, the propensity to study Abstract rather than Practical educational fields is related to socioeconomic instability such as more loneliness and divorce, lower relationship satisfaction, lower vitamin D levels, and higher risks of psychiatric disorders.

Overall, our genetic associations likely pick up on a mixture of causes, consequences, and correlated contexts of educational specialisations, which can be further clarified in future research. We have massively expanded the scope of research on the role of educational fields within the social and life sciences, and generated lots of interesting data and tools for downstream investigation across disciplines.

Did you find the gene or genes for any kind of field specialisation?
No, we did not identify specific genes that directly determine field choices. Our research demonstrates that field specializations, like most complex human behaviours, are influenced by many genetic variants across the genome, each with a very small effect. Rather than finding a few "genes for" field specialization, we discovered polygenic signal—patterns of many genetic variants that collectively show small but measurable associations with different field preferences.

Why are genetic variants associated with fields of education?
The sections above highlighted that genetic influences are always mediated through the environment, and that the Nordic context of the study must be kept in mind when thinking about possible explanatory mechanisms. The figure below brings together the various contexts that individuals are situated in and move through that affect their field of study outcome, all of which end up being captured in genetic studies.

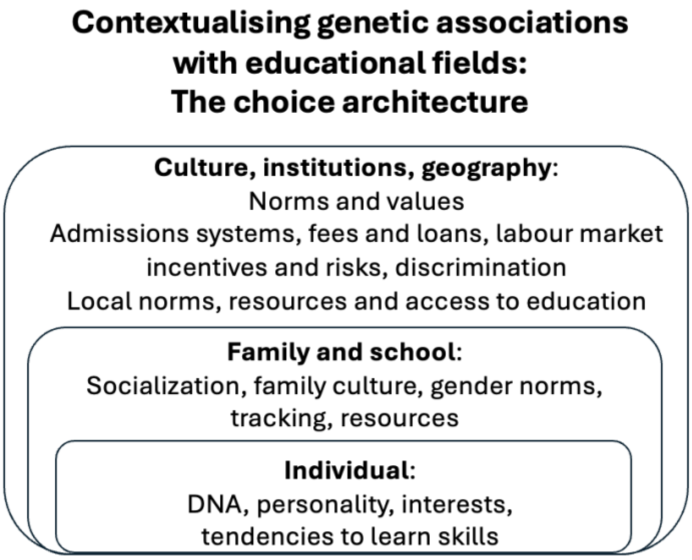

Even in Finland and Norway, educational field choices are constrained and amplified by a multitude of proximal and distal social factors. An individual would not study fine art if they had never heard of fine art or been exposed to encouragement suggesting that it is a suitable choice for them. To the extent that genetic variants contribute to field qualifications, they do so via gene-environment

interplay mechanisms, whereby field-related interests and skills are selected and elicited by individuals based on their heritable traits. Such mechanisms are likely to begin early in life, involving parents, teachers, and other role models. Gender norms are a key social mediator, with stereotypes that influence choice of field of study beginning early. For instance, both girls and boys tend to be steered away from female dominated educational tracks, and the gender gap in STEM degrees is partly because boys benefit from teacher biases. Results could also capture downstream effects of education program prerequisites (e.g., if technical skills are necessary to gain entry to engineering training, then engineers will on average have higher genetic values for technical skills) and pick up dropout due to poor person-environment fit or discrimination. In conclusion, our results reflect the interplay between individual tendencies, social norms and barriers affecting educational qualifications.

Are there any practical uses of genetic results for individuals and policymakers?
An exciting advance in recent years has been the development of individual-level measures of genetic predisposition to traits and diseases – polygenic indices (PGIs). In our study, we show that the genetic association results can be used to create PGIs for specific fields of study in a completely independent Dutch cohort. We saw that participants' PGIs for fields were significantly associated with their actual field of study.

Could PGIs be used to help us make educational decisions? It has been argued that PGIs could provide early warnings for dyslexia. Many of us would be keen to know any information that helps match us with an educational programme.

However, there are technical issues to consider. First, PGI prediction accuracy is extremely weak. Even the strongest PGI predictor, the Arts and humanities PGI, was associated with a miniscule change in log odds for studying Arts and humanities (0.22). Second, we have not even begun to develop PGIs for educational fields in non-European people. Since polygenic Indices (PGIs) derived from one ancestry group cannot be reliably applied to another group, we risk exacerbating existing socioeconomic and health inequalities if these European-derived results are implemented in practice. Our findings show diminished accuracy for individuals with lower genetic similarity to commonly used genomic training sets. These training sets predominantly comprise samples from individuals with recent European ancestry, reflecting a persistent limitation in genomic research. Third, as we have described in the sections above, our genetic association results do not only capture individual interests and skills but a host of other contextual factors like family resources and gender norms. If PGIs are not just personal but circumstantial, this is another way in which using them in practice could exacerbate socioeconomic disparities. To try to establish causality, we conducted analyses controlling for birthplace and parents' education, and we also tested the accuracy of our PGIs within sibling pairs. Although we did not see any strong evidence that PGIs were capturing family environmental and geographical processes, we did not have the statistical power to rule this out completely.

Even if the technical issues were solved and we had PGIs that offered unbiased prediction of our fields of study across ancestries, PGIs cannot capture important individual and contextual information on actual interests, skills, and opportunities. A person with a high polygenic index for technical fields might not prefer to study a technical subject, have the option to pursue it, or be most successful or happy in studying it.

Importantly, we cannot ignore that fields have different normative value and are rewarded differently in the labour market. Even in Norway where many different educational pathways can lead to decent earnings and a good life, health workers tend to earn less than engineers. Using PGIs to inform educational decisions could lead to harmful labelling. Here, it is useful to consider the

consequences of precision/stratified education *without* genetics. Educational tracking, sorting and grouping processes tend to favour socioeconomically advantaged children.

In light of these technical and societal issues, it is difficult and inadvisable to draw conclusions for any individual or for policy based on our genetic study of educational fields.

Does this study show that an individual's choice of educational field is determined, or fixed, at conception? Do genes determine the choices we make and who we become?
Our study does not support any form of genetic determinism regarding educational field choices. Rather, our findings indicate that genetic factors represent just one of many influences linked to educational preferences and field specialization. These genetic influences likely operate through complex, indirect pathways involving cognitive proclivities, personality traits, and interests that develop through continuous interaction with environmental factors. The modest genetic associations we identified explain only a small fraction of the variation in field choices. Most of the variation likely comes from social, cultural, and unique individual experiential factors that support or block certain educational pathways.

What does your study not mean?
Our study does not indicate that genes predetermine or constrain educational or career paths. The polygenic signals we identified should not be interpreted as revealing "genes for" particular fields or suggesting biological essentialism regarding academic or career aptitudes. Furthermore, our findings do not support using genetic information for educational tracking, career counselling, or admissions decisions, as such applications would be scientifically unfounded and ethically problematic. The polygenic indices in our study have limited predictive power at the individual level and only capture statistical tendencies across large populations. Crucially, our results pertain specifically to populations with European genetic ancestry and cannot be generalised to other ancestry groups due to methodological limitations in current genomic research practices.

What has been done to prevent the potential harms of this research?
We have taken multiple steps to mitigate potential misinterpretation and misuse of our findings. First, we explicitly acknowledge the study's limitations, particularly regarding the European ancestry focus of our samples, and emphasize the dangers of extrapolating these results to other populations. Second, we have developed this comprehensive FAQ to address common misconceptions about genetic influences on complex traits. Third, we have engaged with scholars across disciplines, including sociology, to interrogate the implications of our work. Fourth, we are committed to data sharing practices that enable appropriate scientific scrutiny while protecting participant privacy. Finally, we explicitly discourage practical applications of these findings.

Supplementary Note: Ethical Approvals

This study complies with all relevant ethical regulations. The establishment of MoBa and initial data collection was based on a licence from the Norwegian Data Protection Agency and approval from The Regional Committees for Medical and Health Research Ethics. The MoBa cohort is now based on regulations related to the Norwegian Health Registry Act. The Norwegian registry and MoBa data used was from the project SUBPU. The Department of Psychology, University of Oslo, is responsible for the data handling of SUBPU, a Data Protection Impact Assessment (DPIA) has been signed by the head of department, and the project manager is Eivind Ystrom. SUBPU is approved by Committees for Medical and Health Research Ethics (2017/2205). SUBPU has agreements with the MoBa and Statistics Norway for data linkage and usage. The data access and management costs of SUBPU is financed by the Research Council of Norway (RCN) (336078, 288083, and 314601), the European Research Council (101045526, 818425, 101088481, and 818420), the Jacobs Foundation (2023-1510-00), and supported by the Department of Psychology (UiO). All data management and analyses were on the secure data "Tjeneste for Sensitive Data" (TSD) facilities, owned by the University of Oslo.

Study subjects in FinnGen provided informed consent for biobank research, based on the Finnish Biobank Act. Alternatively, separate research cohorts, collected prior the Finnish Biobank Act came into effect (in September 2013) and start of FinnGen (August 2017), were collected based on study-specific consents and later transferred to the Finnish biobanks after approval by Fimea (Finnish Medicines Agency), the National Supervisory Authority for Welfare and Health. Recruitment protocols followed the biobank protocols approved by Fimea. The Coordinating Ethics Committee of the Hospital District of Helsinki and Uusimaa (HUS) statement number for the FinnGen study is Nr HUS/990/2017.

The FinnGen study is approved by Finnish Institute for Health and Welfare (permit numbers: THL/2031/6.02.00/2017, THL/1101/5.05.00/2017, THL/341/6.02.00/2018, THL/2222/6.02.00/2018, THL/283/6.02.00/2019, THL/1721/5.05.00/2019 and THL/1524/5.05.00/2020), Digital and population data service agency (permit numbers: VRK43431/2017-3, VRK/6909/2018-3, VRK/4415/2019-3), the Social Insurance Institution (permit numbers: KELA 58/522/2017, KELA 131/522/2018, KELA 70/522/2019, KELA 98/522/2019, KELA 134/522/2019, KELA 138/522/2019, KELA 2/522/2020, KELA 16/522/2020), Findata permit numbers THL/2364/14.02/2020, THL/4055/14.06.00/2020, THL/3433/14.06.00/2020, THL/4432/14.06/2020, THL/5189/14.06/2020, THL/5894/14.06.00/2020, THL/6619/14.06.00/2020, THL/209/14.06.00/2021, THL/688/14.06.00/2021, THL/1284/14.06.00/2021, THL/1965/14.06.00/2021, THL/5546/14.02.00/2020, THL/2658/14.06.00/2021, THL/4235/14.06.00/2021, Statistics Finland (permit numbers: TK-53-1041-17 and TK/143/07.03.00/2020 (earlier TK-53-90-20) TK/1735/07.03.00/2021, TK/3112/07.03.00/2021) and Finnish Registry for Kidney Diseases permission/extract from the meeting minutes on 4th July 2019.

The Biobank Access Decisions for FinnGen samples and data utilized in FinnGen Data Freeze 11 include: THL Biobank BB2017_55, BB2017_111, BB2018_19, BB_2018_34, BB_2018_67, BB2018_71, BB2019_7, BB2019_8, BB2019_26, BB2020_1, BB2021_65, Finnish Red Cross Blood Service Biobank 7.12.2017, Helsinki Biobank HUS/359/2017, HUS/248/2020, HUS/430/2021 §28, §29, HUS/150/2022 §12, §13, §14, §15, §16, §17, §18, §23, §58 and §59, Auria Biobank AB17-5154 and amendment #1 (August 17 2020) and amendments BB_2021-0140, BB_2021-0156 (August 26 2021, Feb 2 2022), BB_2021-0169, BB_2021-0179, BB_2021-0161, AB20-5926 and amendment #1 (April 23 2020) and it´s modification (Sep 22 2021), BB_2022-0262, BB_2022-0256, Biobank Borealis of Northern Finland_2017_1013, 2021_5010, 2021_5018, 2021_5015, 2021_5015 Amendment, 2021_5023, 2021_5023 Amendment, 2021_5017, 2022_6001, 2022_6006 Amendment, BB22-0067, 2022_0262, Biobank of Eastern Finland 1186/2018 and amendment 22§/2020, 53§/2021, 13§/2022, 14§/2022, 15§/2022, 27§/2022, 28§/2022, 29§/2022, 33§/2022, 35§/2022, 36§/2022, 37§/2022, 39§/2022,

7§/2023, Finnish Clinical Biobank Tampere MH0004 and amendments (21.02.2020 & 06.10.2020), 8§/2021, 9§/2021, §9/2022, §10/2022, §12/2022, 13§/2022, §20/2022, §21/2022, §22/2022, §23/2022, 28§/2022, 29§/2022, 30§/2022, 31§/2022, 32§/2022, 38§/2022, 40§/2022, 42§/2022, 1§/2023, Central Finland Biobank 1-2017, BB_2021-0161, BB_2021-0169, BB_2021-0179, BB_2021-0170, BB_2022-0256, and Terveystalo Biobank STB 2018001 and amendment 25th Aug 2020, Finnish Hematological Registry and Clinical Biobank decision 18th June 2021, Arctic biobank P0844: ARC_2021_1001.

The Lifelines protocol was approved by the UMCG Medical ethical committee under number 2007/152.
