# Supplementary material for: Genetic associations with educational fields
Source: Nat Genet. 2025 Nov 4;57(12):2997–3006. doi: 10.1038/s41588-025-02391-z (PMC12695639; doi:10.1038/s41588-025-02391-z)
Supplement: Supplementary file 2 — Reporting Summary [file 41588_2025_2391_MOESM2_ESM.pdf]

nature portfolio

Corresponding author(s): Rosa Cheesman

Last updated by author(s): 06/03/2025

Reporting Summary

Nature Portfolio wishes to improve the reproducibility of the work that we publish. This form provides structure for consistency and transparency in reporting. For further information on Nature Portfolio policies, see our Editorial Policies and the Editorial Policy Checklist.

Statistics

For all statistical analyses, confirm that the following items are present in the figure legend, table legend, main text, or Methods section.

n/a	Confirmed	
☐	☒	The exact sample size (*n*) for each experimental group/condition, given as a discrete number and unit of measurement
☐	☒	A statement on whether measurements were taken from distinct samples or whether the same sample was measured repeatedly
☐	☒	The statistical test(s) used AND whether they are one- or two-sided<br>*Only common tests should be described solely by name; describe more complex techniques in the Methods section.*
☐	☒	A description of all covariates tested
☐	☒	A description of any assumptions or corrections, such as tests of normality and adjustment for multiple comparisons
☐	☒	A full description of the statistical parameters including central tendency (e.g. means) or other basic estimates (e.g. regression coefficient) AND variation (e.g. standard deviation) or associated estimates of uncertainty (e.g. confidence intervals)
☐	☒	For null hypothesis testing, the test statistic (e.g. *F*, *t*, *r*) with confidence intervals, effect sizes, degrees of freedom and *P* value noted<br>*Give P values as exact values whenever suitable.*
☒	☐	For Bayesian analysis, information on the choice of priors and Markov chain Monte Carlo settings
☒	☐	For hierarchical and complex designs, identification of the appropriate level for tests and full reporting of outcomes
☐	☒	Estimates of effect sizes (e.g. Cohen's *d*, Pearson's *r*), indicating how they were calculated

Our web collection on statistics for biologists contains articles on many of the points above.

Software and code

Policy information about availability of computer code

Data collection	No software was used for data collection.
Data analysis	GWA were performed using FastGWA and REGENIE software. METAL was used to perform meta-analyses. To identify independent genome-wide significant associations in the meta-analytic results, we performed clumping using standard parameters in FUMA software. SNP heritability was esitmated using LD score regression software. Genetic corelations and factor analyses were performed in GenomicSEM R package. PGIs were constructed using SBayesR. Versions: SBayesR within GCTB software version 2.05beta_Linux; gcta_1.91.7beta/gcta64; regenie 2.2.4; Genomic SEM v 0.0.3f in R-4.3.2; METAL for Linux current version 2011-03-25. The code used in this study are available at: https://github.com/rosacheesman/Fields_genetics.

For manuscripts utilizing custom algorithms or software that are central to the research but not yet described in published literature, software must be made available to editors and reviewers. We strongly encourage code deposition in a community repository (e.g. GitHub). See the Nature Portfolio guidelines for submitting code & software for further information.

Data

Policy information about availability of data

All manuscripts must include a data availability statement. This statement should provide the following information, where applicable:

- Accession codes, unique identifiers, or web links for publicly available datasets
- A description of any restrictions on data availability
- For clinical datasets or third party data, please ensure that the statement adheres to our policy

Individual level data: The Finnish biobank data can be accessed through the Fingenious® services (https://site.fingenious.fi/en/) managed by FINBB. Finnish Health register data can be applied from Findata (https://findata.fi/en/data/). Instructions for access to MoBa data from the Norwegian Institute of Public Health can be found here: https://www.fhi.no/en/studies/moba/for-forskere-artikler/research-and-data-access/. Instructions for access to the Lifelines Cohort Study can be found here: https://www.lifelines-biobank.com/researchers/working-with-us. Administrative data on the Dutch population for the purpose of academic research is housed by Statistics Netherlands. Access is only possible for authorised instutions. Instructions on how to apply for the usage of administrative data can be found here: https://www.cbs.nl/en-gb/our-services/customised-services-microdata/microdata-conducting-your-own-research.

Summary statistics: The GWAS summary statistics produced from this study are available at: https://zenodo.org/records/15584414; DOI: 10.5281/zenodo.15584414. Summary statistics from each data FinnGen release will be made publicly available after a one-year embargo period and can be accessed freely from: www.finngen.fi/en/access_results.

Research involving human participants, their data, or biological material

Policy information about studies with human participants or human data. See also policy information about sex, gender (identity/presentation), and sexual orientation and race, ethnicity and racism.

Reporting on sex and gender	We primarily use the term 'sex' because the data were obtained from Norwegian Medical Birth Register e.g., "we conducted sex-stratified GWAS in MoBa (sex defined from birth register data) ".
Reporting on race, ethnicity, or other socially relevant groupings	We primarily use the term 'ancestry' e.g. "European-associated Ancestry individuals " /"To identify a sub-population of European-associated ancestry" since homogenous populations for genetic analysis were identified using allele frequency clustering. We discuss this in the Discussion: "our GWAS were limited to Norwegian and Finnish individuals with European-associated ancestries. It remains unclear how much the results generalise to people of diverse backgrounds. In the future, genetically sensitive studies of educational field choices should include underrepresented groups and countries, taking systematic social differences into account. "
Population characteristics	We use population-wide register data on adults >age 25 from Finland and Norway
Recruitment	We describe this for all cohorts used in the GWAS:<br>"Pregnant women were recruited from across Norway from 1999 to 2009. " for MoBa<br>"Participants were sampled from the northern population of the Netherlands, which is about 10% of the region's population. Between 2006 and 2013, randomly selected general practitioners invited all their listed patients aged 25-49 years to participate in the study" for LifeLines<br><br>"The project utilises data from the nationwide longitudinal health register collected since 1969 from every resident in Finland." for FinnGen
Ethics oversight	The establishment of MoBa and initial data collection was based on a licence from the Norwegian Data Protection Agency and approval from The Regional Committees for Medical and Health Research Ethics. The MoBa cohort is now based on regulations related to the Norwegian Health Registry Act. The current study was approved by The Regional Committees for Medical and Health Research Ethics (project # 2017/2205).<br><br>Study subjects in FinnGen provided informed consent for biobank research, based on the Finnish Biobank Act. Alternatively, separate research cohorts, collected prior the Finnish Biobank Act came into effect (in September 2013) and start of FinnGen (August 2017), were collected based on study-specific consents and later transferred to the Finnish biobanks after approval by Fimea (Finnish Medicines Agency), the National Supervisory Authority for Welfare and Health. Recruitment protocols followed the biobank protocols approved by Fimea. The Coordinating Ethics Committee of the Hospital District of Helsinki and Uusimaa (HUS) statement number for the FinnGen study is Nr HUS/990/2017.<br><br>The FinnGen study is approved by Finnish Institute for Health and Welfare (permit numbers: THL/2031/6.02.00/2017, THL/1101/5.05.00/2017, THL/341/6.02.00/2018, THL/2222/6.02.00/2018, THL/283/6.02.00/2019, THL/1721/5.05.00/2019 and THL/1524/5.05.00/2020), Digital and population data service agency (permit numbers: VRK43431/2017-3, VRK/6909/2018-3, VRK/4415/2019-3), the Social Insurance Institution (permit numbers: KELA 58/522/2017, KELA 131/522/2018, KELA 70/522/2019, KELA 98/522/2019, KELA 134/522/2019, KELA 138/522/2019, KELA 2/522/2020, KELA 16/522/2020), Findata permit numbers THL/2364/14.02/2020, THL/4055/14.06.00/2020, THL/3433/14.06.00/2020, THL/4432/14.06/2020, THL/5189/14.06/2020, THL/5894/14.06.00/2020, THL/6619/14.06.00/2020, THL/209/14.06.00/2021, THL/688/14.06.00/2021, THL/1284/14.06.00/2021, THL/1965/14.06.00/2021, THL/5546/14.02.00/2020, THL/2658/14.06.00/2021, THL/4235/14.06.00/2021, Statistics Finland (permit numbers: TK-53-1041-17 and TK/143/07.03.00/2020 (earlier TK-53-90-20) TK/1735/07.03.00/2021, TK/3112/07.03.00/2021) and Finnish Registry for Kidney Diseases permission/extract from the meeting minutes on 4th July 2019.<br><br>The Biobank Access Decisions for FinnGen samples and data utilized in FinnGen Data Freeze 11 include: THL Biobank

BB2017_55, BB2017_111, BB2018_19, BB_2018_34, BB_2018_67, BB2018_71, BB2019_7, BB2019_8, BB2019_26, BB2020_1, BB2021_65, Finnish Red Cross Blood Service Biobank 7.12.2017, Helsinki Biobank HUS/359/2017, HUS/248/2020, HUS/430/2021 §28, §29, HUS/150/2022 §12, §13, §14, §15, §16, §17, §18, §23, §58 and §59, Auria Biobank AB17-5154 and amendment #1 (August 17 2020) and amendments BB_2021-0140, BB_2021-0156 (August 26 2021, Feb 2 2022), BB_2021-0169, BB_2021-0179, BB_2021-0161, AB20-5926 and amendment #1 (April 23 2020) and it´s modification (Sep 22 2021), BB_2022-0262, BB_2022-0256, Biobank Borealis of Northern Finland_2017_1013, 2021_5010, 2021_5018, 2021_5015, 2021_5015 Amendment, 2021_5023, 2021_5023 Amendment, 2021_5017, 2022_6001, 2022_6006 Amendment, BB22-0067, 2022_0262, Biobank of Eastern Finland 1186/2018 and amendment 22§/2020, 53§/2021, 13§/2022, 14§/2022, 15§/2022, 27§/2022, 28§/2022, 29§/2022, 33§/2022, 35§/2022, 36§/2022, 37§/2022, 39§/2022, 7§/2023, Finnish Clinical Biobank Tampere MH0004 and amendments (21.02.2020 & 06.10.2020), 8§/2021, 9§/2021, §9/2022, §10/2022, §12/2022, 13§/2022, §20/2022, §21/2022, §22/2022, §23/2022, 28§/2022, 29§/2022, 30§/2022, 31§/2022, 32§/2022, 38§/2022, 40§/2022, 42§/2022, 1§/2023, Central Finland Biobank 1-2017, BB_2021-0161, BB_2021-0169, BB_2021-0179, BB_2021-0170, BB_2022-0256, and Terveystalo Biobank STB 2018001 and amendment 25th Aug 2020, Finnish Hematological Registry and Clinical Biobank decision 18th June 2021, Arctic biobank P0844: ARC_2021_1001.

The Lifelines protocol was approved by the UMCG Medical ethical committee under number 2007/152.

Note that full information on the approval of the study protocol must also be provided in the manuscript.

Field-specific reporting

Please select the one below that is the best fit for your research. If you are not sure, read the appropriate sections before making your selection.

☐ Life sciences ☒ Behavioural & social sciences ☐ Ecological, evolutionary & environmental sciences

For a reference copy of the document with all sections, see nature.com/documents/nr-reporting-summary-flat.pdf

Behavioural & social sciences study design

All studies must disclose on these points even when the disclosure is negative.

Study description	Quantitative research - GenomeWide Association Meta-analysis of Educational Fields.
Research sample	We required the maximum sample of adults (age 25) with educational records AND genotype data.This meant using Finnish and Norwegian data from educational registries linked to FinnGen and MoBa. Administrative data are of high quality, and do not suffer attrition. Our study only loses representativeness by being restricted to genotyped cohort study participants of European ancestry (necessary to avoid population stratification). The sample is wealthier and healthier than the general populations.
Sampling strategy	Our strategy was to collect the maximum sample of participants from European-associated ancestries with genotype and educational phenotype information. Our sample size of >460,000 meets power requirements for identifying SNP associations, and Polygenic Index analyses include well over the minimum of several hundred participants. FinnGen used population-wide registers, MoBa convenience sampling of pregnant families, and Lifelines stratified random sampling via general practitioners.
Data collection	We used previously collected data, with collection and recruitment described elsewhere. Phenotypes used were recorded through linked administrative data. Educational field data were extracted from administrative registers using national systems—Statistics Finland, Norwegian Standard Classification of Education (NUS2000) in MoBa, and Statistics Netherlands administrative files ("HOOGSTEOPLTAB 2022, V1") in Lifelines—then harmonized to ISCED 2013 broad field categories. Admin records are described here: https://www2.stat.fi/fi/luokitukset/koulutusala/ and: http://www.ssb.no/en/utdanning/norwegian-standard-classification-of-education. Data collection was prior to initiation of this study. The current analysts were not blinded to study hypotheses.
Timing	Education register data includes entire populations year-by-year. We used most recently available data available in both (2018) on adults aged >25. Data collection spanned 06/1999-12/2009 (MoBa), 01/2006-12/2013 (Lifelines), and 01/1969-ongoing (FinnGen administrative data, genetic collection from 2017).
Data exclusions	We excluded participants for missingness on phenotypic and genomic variables of study. A total of 5, 601, 162 individuals with educational field data were excluded (2, 857, 143 from Norway and 2, 744, 019 from Finland) due to lack of genetic data.
Non-participation	Not applicable - -- administrative registers include the total populations. However, not all initial MoBa and FinnGen participants consented to genotyping.
Randomization	Not applicable, participants were not allocated into experimental groups.

Reporting for specific materials, systems and methods

We require information from authors about some types of materials, experimental systems and methods used in many studies. Here, indicate whether each material, system or method listed is relevant to your study. If you are not sure if a list item applies to your research, read the appropriate section before selecting a response.

Materials & experimental systems

n/a	Involved in the study
☒	☐ Antibodies
☒	☐ Eukaryotic cell lines
☒	☐ Palaeontology and archaeology
☒	☐ Animals and other organisms
☒	☐ Clinical data
☒	☐ Dual use research of concern
☒	☐ Plants

Methods

n/a	Involved in the study
☒	☐ ChIP-seq
☒	☐ Flow cytometry
☒	☐ MRI-based neuroimaging

Plants

Seed stocks	*Report on the source of all seed stocks or other plant material used. If applicable, state the seed stock centre and catalogue number. If plant specimens were collected from the field, describe the collection location, date and sampling procedures.*
Novel plant genotypes	*Describe the methods by which all novel plant genotypes were produced. This includes those generated by transgenic approaches, gene editing, chemical/radiation-based mutagenesis and hybridization. For transgenic lines, describe the transformation method, the number of independent lines analyzed and the generation upon which experiments were performed. For gene-edited lines, describe the editor used, the endogenous sequence targeted for editing, the targeting guide RNA sequence (if applicable) and how the editor was applied.*
Authentication	*Describe any authentication procedures for each seed stock used or novel genotype generated. Describe any experiments used to assess the effect of a mutation and, where applicable, how potential secondary effects (e.g. second site T-DNA insertions, mosiacism, off-target gene editing) were examined.*
